# Convention of American Medical Editors

**Published:** 1877-07

**Authors:** 


### CONVENTION OF AMERICAN MEDICAL EDITORS.

The Association of American Medical Editors met in the reading-room of the Palmer House, on the evening of June 4th. H. C. Wood, M.D., of Philadelphia, occupied the chair, and Dr. F. H. Davis, of Chicago, acted as secretary.

The President, in his annual address, threw out the following points for discussion: 1. The number of medical periodicals in this country is too large; a reduction in number would afford a few really good journals a decent support; 2. Societies should be induced to discontinue the printing of their transactions in extra volumes which reach but a very limited number of readers; those transactions should be carefully condensed and then published in some medical journal which the society could afford to send to every member for less than the cost of the annual publication of the transactions. Under this arrangement the members of the society and the journal would be benefitted; the former by receiving a medical journal gratis, and the latter by swelling its subscription list. 3. It is time that the medical colleges improve their method of teaching and elevate the standard of preliminary education. 4. Some means of protecting the people and the profession against quackery is urgently needed; perhaps the best plan would be to have a State Board of Examiners to examine candidates in these branches which are common to all schools, being supplemented by boards of therapeutists of the several schools to examine in those subjects on which the schools differ.

After a brief discussion upon the President's address the election of officers for the ensuing year was in order.

Dr. John P. Gray, of Utica, was elected President, and Dr. L. Connor, of Detroit, Vice-President. Dr. F. H. Davis, Secretary, held over.

The Association then adjourned until next year.—*Journal and Examiner.*

